# Achieving malaria elimination and certification in Turkmenistan

**DOI:** 10.1186/1475-2875-11-S1-O11

**Published:** 2012-10-15

**Authors:** Leili Shamuradova, Sofia Alieva, Rossitza Kurdova-Mintcheva, Aafje Rietveld, Richard Cibulskis, Mikhail Ejov, Bahtygul Karrieva, Robert D Newman

**Affiliations:** 1 Ministry of Health, Ashgabat, Turkmenistan; 2 Global Malaria Programme, World Health Organization, Geneva, Switzerland; 3 WHO Regional Office for Europe, Copenhagen, Denmark; 4 WHO country Office, Ashgabat, Turkmenistan

## 

This abstract is submitted as part of the panel session on case studies for elimination by the WHO Global Malaria Programme and the UCSF Global Health Group.

## Background

This case study presents and evaluates the strategies and policies applied for containment of re-emerging malaria outbreaks in Turkmenistan since the 1990s, and the process followed for achieving malaria elimination. Evidence-based lessons for countries that are considering or embarking upon elimination are distilled. The case study is a part of a series of malaria elimination case studies conducted by the WHO Global Malaria Programme and the University of California, San Francisco, Global Health Group. Key partners in the case study work were the National Malaria Control Programme, Ministry of Health, and WHO Regional and Country Offices.

## Materials and methods

A comprehensive search was made of English and Russian language materials related to malaria in Turkmenistan, as well as selected materials in Turkmen language. Published and grey literature; National Malaria Programme data and other official data from the Ministry of Health; information from direct observations; and WHO archives were consulted. Epidemiological, programmatic, social and economical determinants were extracted and analysed to evaluate (1) the malaria epidemiological situation; (2) social and economical factors that influence malaria situation; and (3) programme operations.

## Results

Turkmenistan achieved malaria elimination by the 1960s, after which only sporadic imported and introduced cases of *Plasmoidum vivax* were reported. In the 1980s and 1990s the malaria threat increased due to rising receptivity in some areas (owing to major water projects such as construction of the Karakum Canal, expanded irrigation, and increased rice production) , as well as to increasing vulnerability in districts bordering Afghanistan related to growing population movement. Malaria importation from Afghanistan increased, followed by an increase of autochthonous cases. Outbreaks of vivax malaria were registered in Mary province on the border with Afghanistan in 1998-99 (in a military training camp) and in 2002-03 (among petroleum workers). The main interventions to contain the outbreaks were: intensive case detection (daily house-to-house visits; mass blood survey) with subsequent treatment of those found to be positive; epidemiological investigation of all cases and foci; radical treatment of patients; IRS; and larviciding. The population in active transmission foci received seasonal chemoprophylaxis with chloroquine and after-season radical treatment with 14 days of primaquine.

Following the outbreaks, malaria elimination was achieved by: (1) strong political commitment and sustained national funding; (2) introduction of a National Strategy and Plan of Action for Malaria Elimination; (3) case-based surveillance of confirmed cases through quality-assured laboratories; (4) integrated vector control in foci; and (5) cross-border collaboration with Afghanistan. The last autochthonous cases were registered in 2004. In 2010, the country was certified by WHO as free of malaria.

## Conclusions

The Turkmenistan case study is an example of the correct application of evidence-based malaria elimination strategies and policies to overcome a reintroduction of malaria transmission and subsequently achieve malaria elimination. It illustrates the strong political commitment, programmatic efforts and substantial funding needed to design and implement a malaria elimination programme. The Turkmenistan experience highlights the importance of a clear plan for prevention of malaria reintroduction following elimination, and the need for continued financial resources to execute these critical activities.

